# Inverse Correlation between Heart Rate Variability and Heart Rate Demonstrated by Linear and Nonlinear Analysis

**DOI:** 10.1371/journal.pone.0157557

**Published:** 2016-06-23

**Authors:** Syed Zaki Hassan Kazmi, Henggui Zhang, Wajid Aziz, Oliver Monfredi, Syed Ali Abbas, Saeed Arif Shah, Syeda Sobia Hassan Kazmi, Wasi Haider Butt

**Affiliations:** 1 School of Physics & Astronomy, University of Manchester, Manchester, United Kingdom; 2 Department of CS&IT, University of Azad Jammu and Kashmir, Muzaffarabad, Pakistan; 3 College of Electrical and Mechanical Engineering, National University of Sciences and Technology, Rawalpindi, Pakistan; 4 Department of Computer Science Faculty of Computing &IT, University of Jeddah, Jeddah, Kingdom of Saudi Arabia; 5 Institute of Cardiovascular Sciences, University of Manchester, Manchester, United Kingdom; Centro Cardiologico Monzino, ITALY

## Abstract

The dynamical fluctuations in the rhythms of biological systems provide valuable information about the underlying functioning of these systems. During the past few decades analysis of cardiac function based on the heart rate variability (HRV; variation in R wave to R wave intervals) has attracted great attention, resulting in more than 17000-publications (PubMed list). However, it is still controversial about the underling mechanisms of HRV. In this study, we performed both linear (time domain and frequency domain) and nonlinear analysis of HRV data acquired from humans and animals to identify the relationship between HRV and heart rate (HR). The HRV data consists of the following groups: (a) human normal sinus rhythm (n = 72); (b) human congestive heart failure (n = 44); (c) rabbit sinoatrial node cells (SANC; n = 67); (d) conscious rat (n = 11). In both human and animal data at variant pathological conditions, both linear and nonlinear analysis techniques showed an inverse correlation between HRV and HR, supporting the concept that HRV is dependent on HR, and therefore, HRV cannot be used in an ordinary manner to analyse autonomic nerve activity of a heart.

## Introduction

Greek Physicians and scientists were the pioneers who measured pulse rate; however, it was not much accurate till the invention of pulse watch in 1707 [1]. The correlation between heart rate variability, blood pressure and respiratory rate was first observed by Stephen Hales in 1733 [1]. His observation was confirmed by Carl Ludwig in 1847[1], who noted increase in heart rate and blood pressure with inspiration and a decrease in these two parameters with expiration. The advances in digital signal processing techniques and long term ambulatory ECG recording opened a new arena for HRV analysis and its linkages to health and disease [1]. The study of heart rate variability (HRV) is a research area that has attracted more and more researchers, causing significant increase in the number of publications in this field. According to PubMed present list of publications in this research area exceeds 17000 research articles [2].

The autonomic nervous system (ANS) controls heart rate, hear rate variability (HRV) and breathing. Heart Rate Variability (HRV) analysis is a non-invasive technique used to evaluate the balancing action of sympathetic and parasympathetic branches of the autonomic nervous system [3, 4,5]. Over the last four decades advances in the data acquisition systems and their associated computational tools have resulted into fast and robust application methods to extract valuable information from heart rate signals. HRV reflects the cardiac autonomic control of the ANS and its measurement can provide additional information about the controlling mechanism of ANS compared to the measurements of heart rate alone.

Over the last 35 years, various linear and non-linear techniques have been developed to extract the valuable information from cardiac interbeat interval time series data, aiming to help clinician for prognostication of illness and assessing malfunctioning of the autonomic nervous system. However, often contradictory results have left clinicians skeptical and there exists no clear consensus about HRV analysis measures [6]. In 1996, the Task force of the ESC/NASPE published standards in the measurement, interpretation and clinical use of HRV in cardiology [7]. The time domain measures are predominantly being used for long term profile of HRV and frequency domain HRV measures are being used for analysis of short term HRV data [7]. Heart is not a periodic oscillator under normal physiologic conditions [7] and standard time and frequency domain HRV measures may not be able to transient changes in the RR-interval time series data. The use of nonlinear measures may lead to substantial improvements in understanding transient changes in heart period and their physiological and pathophysiological correlates [7, 8]. The HRV analysis have been used in investigating wide spectrum of cardiac and non-cardiac disease, however, its practical use in adult medicine has been reached in two clinical scenarios only. The reduced HRV has been attributed to the risk stratification after acute myocardial infarction (MI) and as an early warning sign of diabetic neuropathy.

The main purpose of this study is to identify the relationship of HR with both linear and nonlinear metrics of HRV. The human and animal data sets were analysed under following conditions: (a) human normal sinus rhythm (n = 72); (b) human congestive heart failure (n = 44); (c) rabbit sinoatrial node cell (SANC) (n = 67); and (d) conscious rat (n = 11). Both nonlinear and linear methods showed that HRV is primarily dependent on HR. There is an inverse correlation between the HRV and HR: a larger HRV (R-R interval) was correlated with a lower HR, and the vice versa. Such a correlation was observed both in the human data at variant pathological conditions and variant animal species. This study provided support to the concept of a previous study[2] on that HRV cannot not be simply used as a biological marker for cardiac autonomic nervous system activity, and the association between a variation in HRV and altered mortality and coronary heart disease may be significantly attributable to the variation in HR [2,9,10].

## Materials and Methods

### HRV Analysis

HRV analysis can be done by many ways and a wide range list of techniques explored since 1960 are described in detail by the North American Society of Pacing Electrophysiology and the task force for European Society of Cardiology [7]. This study investigated both linear (frequency domains and time domain) and nonlinear HRV parameters.

### Linear HRV techniques

Measures included in linear time domain parameters are derived from direct RR interval measurement for standard deviation of all normal to normal RR intervals (SDNN), and from differences of RR interval for root mean square of successive NN interval differences (RMSSD) (RMSSD) [7]. Specifically, SDNN are calculated as

$$SDNN=\sqrt{\frac{1}{N-1}\sum_{n=2}^{N}{\left[I(n)-\bar{I}\right]}^{2}}$$

In which ‘I’ is RR intervals, $\prime \bar{I\prime }$ is the mean of RR intervals and ‘N’ is the total number of RR intervals. RMSSD are calculated as

$$RMSSD=\sqrt{\frac{1}{N-2}\sum_{n=3}^{N}{\left[I(n)-I(n-1)\right]}^{2}}$$

Where ‘I’ is RR intervals and ‘N’ is the total number of RR intervals.

The frequency domain measures used in this study included: Very low frequency (VLF), low frequency (LF), high frequency (HF) and total power. VLF is a band of power spectrum range ≥ 0.04 Hz. Normally this measure shows overall activity of numerous slow mechanisms of sympathetic activity. LF is a band of power spectrum ranging between 0.04 and 0.15 Hz. This parameter reflects both sympathetic and parasympathetic function. HF is a band of power spectrum range between 0.15 and 0.4 Hz. Generally this measure reflects parasympathetic function. Total power is a short term estimate of the total power of power spectral density in the range of frequencies between 0 and 0.4 Hz. In this parameter sympathetic activity is a main contributor when its shows overall autonomic activity [11].

#### Nonlinear HRV techniques

Approximate entropy (ApEn) and sample entropy (SamEn) were used to analyse the nonlinear behaviour of HRV [12, 13,14]. Unevenness or randomness of the signal can be measured with ApEn, which measures the predictability of the variations in the signal [12]. The length of the vectors (m), and the tolerance (r) are the two factors on which the values of ApEn depend. ApEn is related to the probability that segments of m data samples which are similar (i.e. closer each other than given distance r) remain similar when the segment length increase to m+1. The parameter m is the value of size of vectors for comparison in particular section of RR-intervals. The parameter r can be set as a certain percentage of standard deviation (SD) of original time series (for HRV analysis, r normally ranges from 10 to 25% of SD), the recommended range in various articles. Approximate Entropy can be written as ApEn (m, r). A smaller ApEn value indicates a more regular signal and a larger ApEn value indicate higher complexity of the signal. The main benefit of ApEn measure is that it may be computed for short time series of data. Pincus [12] computed that data of 10^m^ or 30^m^ and m = 2(100–900 data points) will yield statically reproducible and reliable results. He also describe that ApEn can be applied to any system having at least 50 data points. ApEn algorithm counts similar sequences to given sequence of length m, including the sequence itself to avoid natural logarithm of zero within the calculations. As a result, ApEn can sensitive to the size of data. To overcome this, a variant of the ApEn algorithm called Sample entropy (SamEn) [13] was proposed, which excludes self-matches. SamEn has the advantage of being less dependent on the time series length and shows consistency over broad ranges of possible m, r and N (number of data point in the time series). In this study, we used parameters m = 2 and r = 0.2 for computation of ApEn and SamEn. The values of parameters m and r we used were based on the previous studies of Pincus [12], which gave good statistical validity for ApEn calculation.

## Data Sets

The data sets used in the study comprised of both human and animal data and in Table 1 details of the data used are illustrated.

**Table 1:** R-wave to R-wave interval data sets used in the study.

Data Sets	Number of subjects	
Human normal sinus rhythm (NSR)	72	Human normal sinus rhythm data was taken from the two publically available databases comprising 72 subjects (35 men and 37 female) aging between 20 to 78 years. The data sets taken from MIT-BIH Normal Sinus Rhythm Database comprised of 18 hours ECG recordings of 24 subjects. The rest of data comprising of 54 beat annotation files of long term ECG recording was taken from Normal Sinus Rhythm RR-interval database [15].
Human congestive heart failure (CHF)	44	The human congestive heart failure time series data comprising of 44 subjects (15 women and 29 men) was taken from Physionet. The 15 subjects with severe CHF (NYHA class 3–4) were taken from BIDMC Congestive Heart Failure Database. The rest of data was taken from congestive heart failure RR interval database comprising beat annotation files for 29 long-term ECG recordings of subjects with CHF (NYHA classes 1, 2, and 3) [15].
Rabbit SANC	67	Hypertension.2014;64:00–00 [2].
Conscious rat	11	Hypertension.2014;64:00–00 [2].

## Results

### Time Domain Analysis

**Fig 1:**
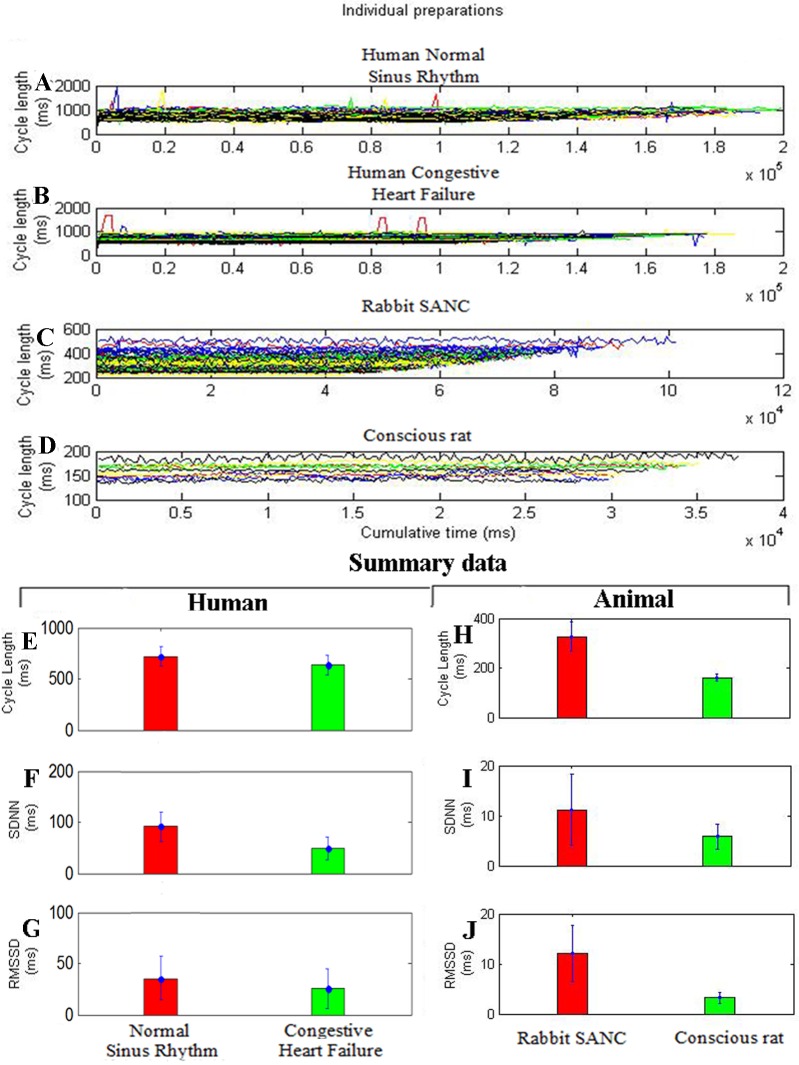
Differences in heart rate variability (HRV) among different cardiac preparations (baseline conditions). A to D, Tachograms from: (a) human normal sinus rhythm (n = 72); (b) human congestive heart failure (n = 44);(c) rabbit SANC(sinoatrial node cell; n = 67);(d) conscious rat(n = 11) subjects. Individual experiments are plotted in unique colours. E to J, Summary of baseline differences in cycle length (CL) and HRV among cardiac preparations. Mean (+SEM) CL (E,H), SD of normal beat to normal beat intervals (SDNN; F,I), and root mean square of successive differences (RMSSD) (G,J) for different preparations.

We used RR-interval time series data of human normal sinus rhythm (n = 72), human congestive heart failure(n = 44), rabbit SANC(sinoatrial node cell; n = 67) and conscious rat(n = 11) subjects.Fig 1A–1D shows tachograms for the different preparations, demonstrating marked differences in HRV under baseline conditions; the data are summarized in Fig 1E–1J.

In human groups, HRV in terms of SDNN and RMSSD is high in normal sinus rhythm (Fig 1A, 1F and 1G) then in congestive heart failure (Fig 1B, 1F and 1G) respectively. Similarly in animal groups, HRV in terms of SDNN and RMSSD is high in rabbit SANC (Fig 1C, 1I and 1J) then conscious rat (Fig 1D, 1I and 1J). The baseline cycle length (CL; same as R-R or NN interval) also varied widely between preparations (Fig 1E and 1H). In human groups preparation with the longest CL is normal sinus rhythm (713±95 ms) then congestive heart failure (657±97 ms) (Fig 1E). Similarly in animal groups preparation with the longest CL is rabbit SANC(326±58 ms) then conscious rat(160±14 ms) (Fig 1H). Below we argue that HRV is strongly dependent on CL: the shorter the CL, the less the HRV. This explains the low HRV(SDNN and RMSSD) in the human congestive heart failure (Fig 1F and 1G) and conscious rat (Fig 1I and 1J), which has the shortest CL (Fig 1F and 1I).

### Frequency Domain Analysis

**Fig 2:**
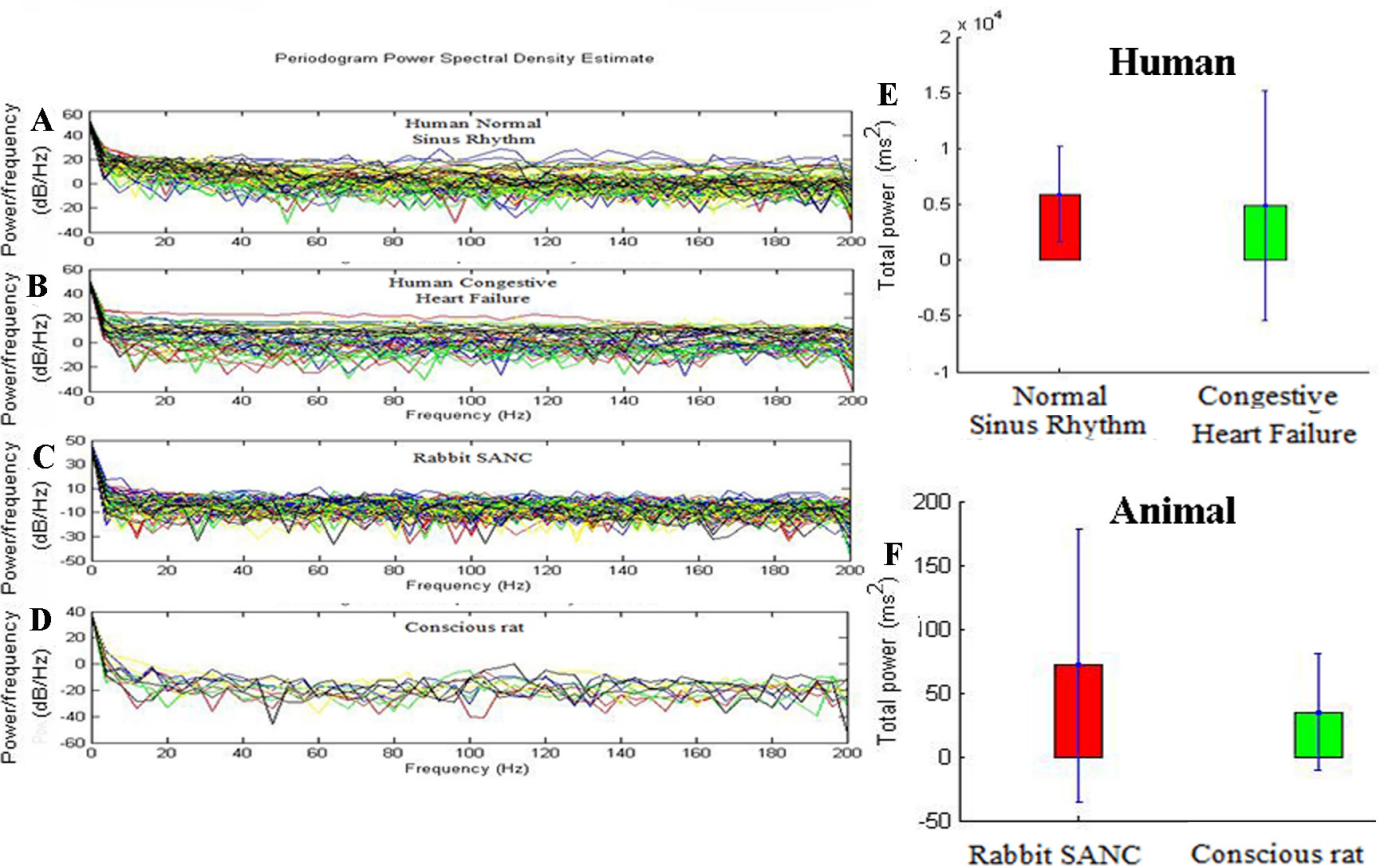
Differences in heart rate variability (HRV) among different cardiac preparations (baseline conditions) using frequency domain analysis. A toD, Tachograms from: (a) human normal sinus rhythm (n = 72); (b) human congestive heart failure (n = 44);(c) rabbit SANC(sinoatrial node cell; n = 67);(d) conscious rat(n = 11) subjects. E and F shows the total power among different cardiac preparations.

In frequency domain analysis we used Total Power(TP) (high frequency (HF)+low frequency (LF)+very low frequency (VLF)) to analyse RR-interval time series data of human normal sinus rhythm (n = 72),human congestive heart failure(n = 44), rabbit SANC(sinoatrial node cell; n = 67) and conscious rat(n = 11). Fig 2A–2D: Power spectra under baseline conditions from all four species studied to show difference in power present at frequencies.

HRV in terms of Total Power in human groups is high in the normal sinus rhythm(Fig 2E) then congestive heart failure (Fig 2E). Similarly, HRV in terms of Total Power in animal groups is high in the rabbit SANC (Fig 2F) then conscious rat (Fig 2F). The baseline cycle length (CL; same as R-R or NN interval) also varied widely between preparations (Fig 1E and 1H). In human groups preparation with the longest CL is normal sinus rhythm (713±95 ms) then congestive heart failure (657±97 ms) (Fig 1E). Similarly in animal groups preparation with the longest CL is rabbit SANC(326±58 ms) then conscious rat(160±14 ms) (Fig 1H). Below we argue that HRV is strongly dependent on CL: the shorter the CL, the less the HRV. This explains the low HRV(Total Power) in the human congestive heart failure (Fig 2E)and conscious rat (Fig 2F), which has the shortest CL (Fig 1E and 1H).

### Nonlinear Time Series Analysis

**Fig 3:**
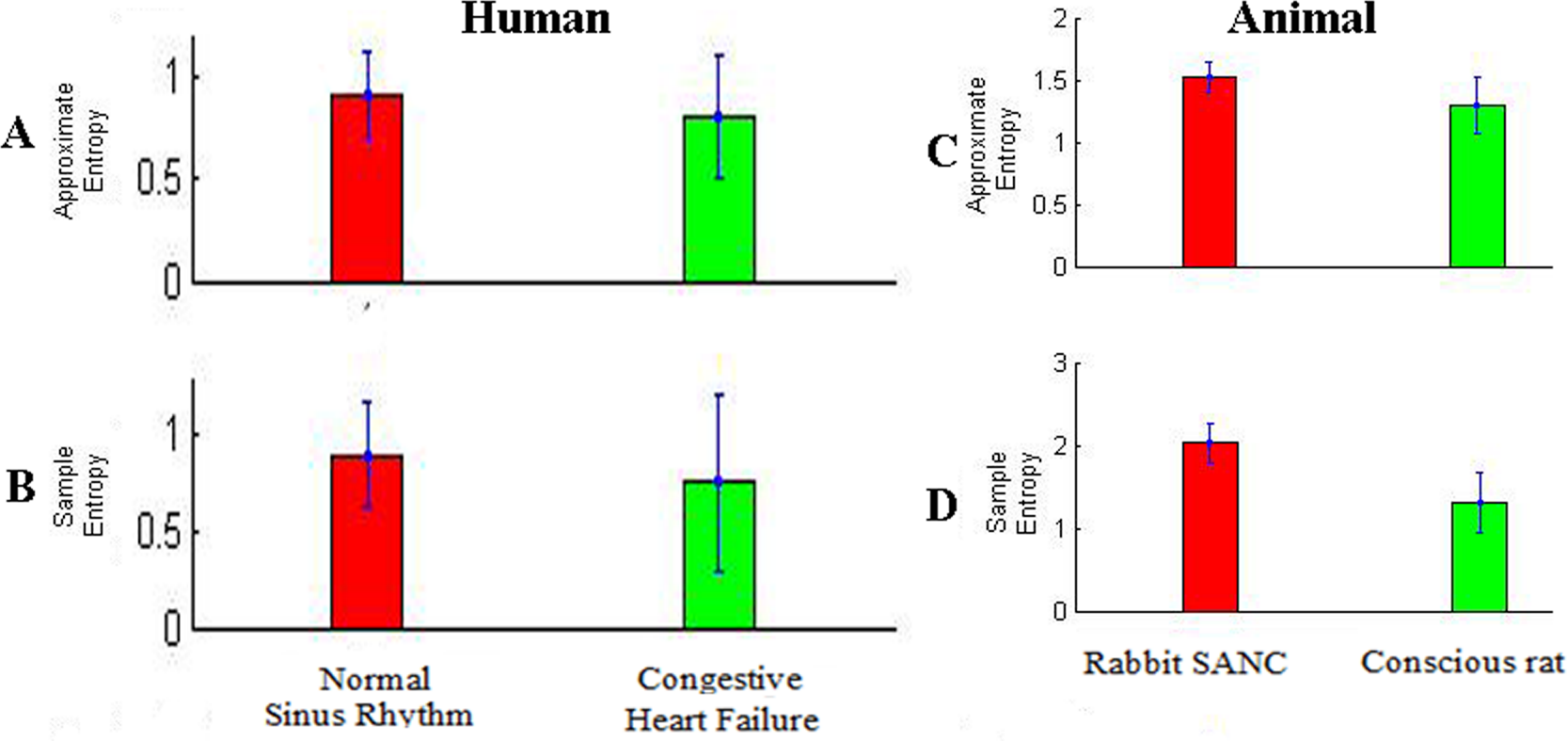
Differences in heart rate variability (HRV) among different cardiac preparations (baseline conditions) using Approximate Entropy and Sample Entropy, a nonlinear time series analysis.

In nonlinear time series analysis we used Approximate Entropy and Sample Entropy to analyse RR-interval time series data of human normal sinus rhythm (n = 72),human congestive heart failure(n = 44), rabbit SANC(sinoatrial node cell; n = 67) and conscious rat(n = 11). In human groups, HRV in terms of Approximate Entropy and Sample Entropy is high in normal sinus rhythm (Fig 3A and 3B) then congestive heart failure (Fig 3A and 3B). Similarly in animal groups, HRV in terms of Approximate Entropy and Sample Entropy is high in rabbit SANC (Fig 3C and 3D) then conscious rat(Fig 3C and 3D).

The baseline cycle length (CL; same as R-R or NN interval) also varied widely between preparations (Fig 1F and 1I). In human groups preparation with the longest CL is normal sinus rhythm (713±95 ms) then congestive heart failure (657±97 ms) (Fig 1E). Similarly in animal groups preparation with the longest CL is rabbit SANC (326±58 ms) then conscious rat(160±14 ms) (Fig 1H). Below we argue that HRV is strongly dependent on CL: the shorter the CL, the less the HRV. This explains the low HRV(Approximate Entropy and Sample Entropy) in the Human Congestive Heart Failure (Fig 3A and 3B) and Conscious Rat (Fig 3C and 3D), which has the shortest CL (Fig 1E and 1H).

### Relationship between HRV and HR

**Fig 4:**
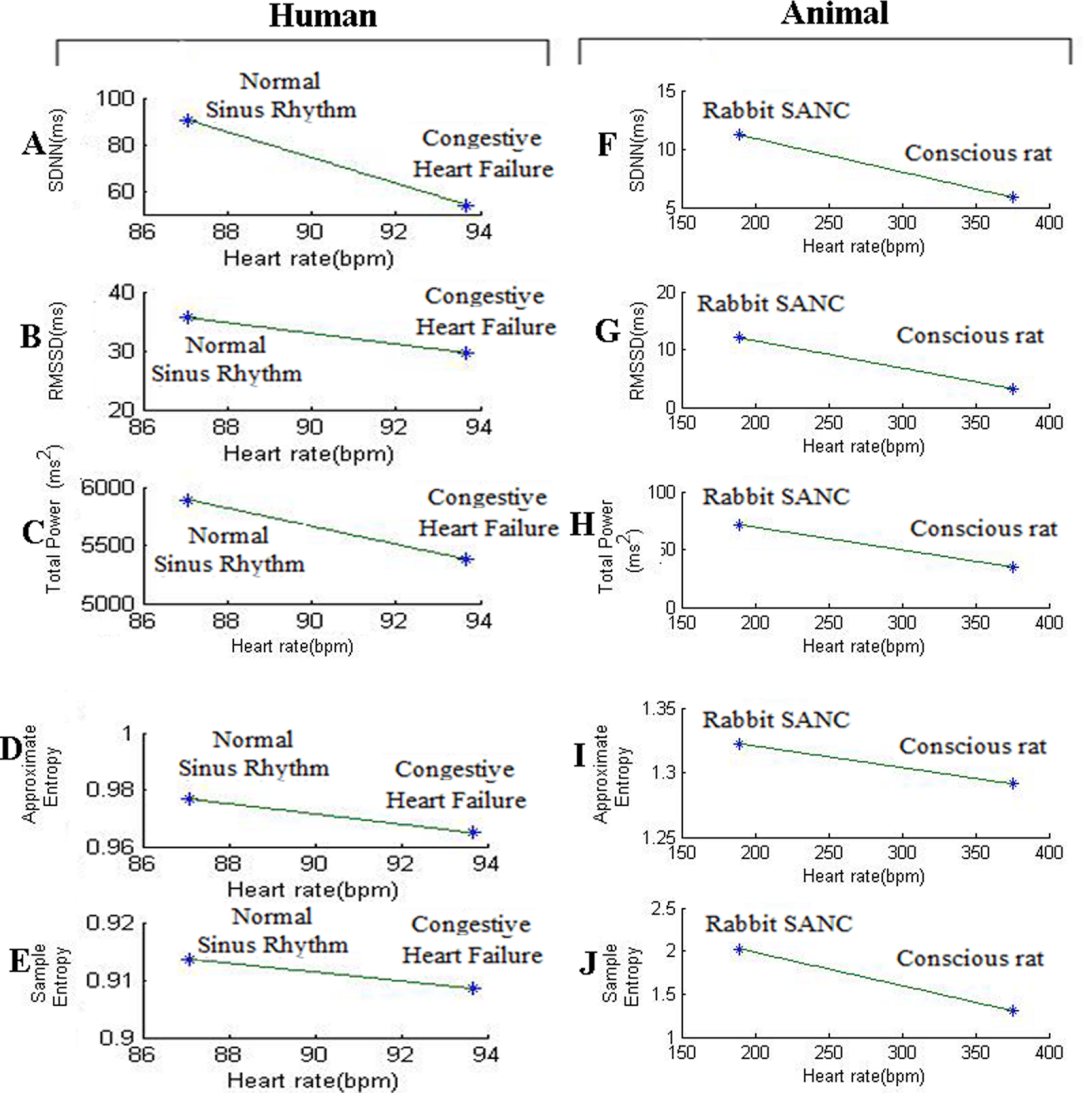
Relationship between HRV metrics (SDNN, RMSSD, total power, approximate entropy, sample entropy) and heart rate.

In the Figs 1 and 3, indirect method has been used to identify the relation between HR and HRV, which first finds the relationships of cycle length (CL) with HRV. HRV is directly proportional to the CL, i.e. with increase in cycle length HRV also increases and vice versa. Since cycle length is inversely proportional to HR, it can be inferred that HRV is inversely proportional to that HR. In the Fig 4, the direct method is used to identify relationship of HR and various linear and nonlinear HRV metrics.

Fig 4A–4E present the results of HRV linear (SDNN, RMSSD and total power) nonlinear (approximate entropy and sample entropy) measures plotted against HR for the human NSR and CHF groups. The results indicated that both linear and nonlinear metrics of HRV showed a decreasing with increasing HR. Similar results were obtained for the animal data as shown in the Fig 4F–4J. Thus the findings clearly demonstrate an inverse relation of HRV metrics with HR using both direct and indirect methods.

## Discussion

Heart rate variability analysis is a non-invasive technique used to assess the cardiac autonomic control under physiological and pathophysiological conditions [3, 4, 7]. The Task force of the ESC/NASPE in 1996 published standards in the measurement, interpretation and clinical use of HRV in cardiology [7]. Numerous studies reported that decreased HRV is associated with cardiovascular morbidity [3, 4, 7]. It has been well established in numerous studies that HR has significant effect on HRV [2, 16, 17]. Mangin et al [16], studied the relationship between HR and HRV parameters (SDNN, RMSSD and sum of LF and HF power). They found that HRV parameters were significantly correlated with cycle length and hence the heart rate. In another study conducted by Coumel et al [17], demonstrated a significant relation SDNN and HR (correlation coefficient 0.79). However, both of these studies did not show the quantitative relationship between HRV and HR.

Monfredi et al [2], used linear HRV parameters (SDNN, RMSSD) from variety of cardiac preparations in diverse species to establish a quantitative relationship between HR and HRV. They found exponetially decreasing trend in HRV with increasing heart rate. By using two biophysical models they confirmed that HRV is primarily dependent on HR and cannot be used to independently assess the cardiac autonomic function. However, the study investigated the relationship of heart rate with linear HRV parameters only. Heart is not a periodic oscillator under normal physiologic conditions [7], the use of nonlinear HRV measures may provide better information to understand transient changes in heart period and their physiological and pathophysiological correlates [7]. In this study, relationship of both linear (SDNN, RMSSD, Total Power) and nonlinear (Approximate Entropy and Sample Entropy) HRV parameters with HR was investigated using indirect and direct methods. In indirect method, we first identified the relationship between cycle length and HRV parameters as shown in Figs 1 and 3, it was found that HRV has directly correlated with cycle length i.e. with increase in cycle length HRV also increased and vice versa. As cycle length is inversely correlated to HR, we concluded that HRV can also be inversely correlated to HR. In direct method we have directly plotted HRV (SDNN, RMSSD, Total Power, Approximate Entropy, Sample Entropy) against HR (Fig 4). The simulations indicate that HRV is primarily dependent on HR. There is an inverse correlation between the HRV and HR: a larger HRV (R-R interval) was correlated with a lower HR, and the vice versa. Such a correlation was observed both in the human data at variant pathological conditions and variant animal species.

## Conclusion

In this study, we investigated the relationship of linear (time domain and frequency domain) and nonlinear HRV parameters with heart rate using RR-interval time series data from human and animals. The RR-interval time series comprised of human (72 NSR and 44 CHF subjects) and animal subjects (67 rabbit sinoatrial node cells data and 11 conscious rats). The research findings indicate that both linear and nonlinear HRV methods showed an inverse correlation between the HRV and HR: a larger HRV (R-R interval) was correlated with a lower HR, and the vice versa. Such a correlation was observed both in the human data at variant pathological conditions and in different animal species. The inverse correlation of nonlinear HRV measures along with linear ones with HR is the salient feature of this study. The outcomes of this study strong the evidence of relationship between HRV with HR and are in line with outcomes of Monfredi and co-authors. It is suggested that in future studies HR should be taken into consideration at which HRV analysis is being performed.
